# Infant Born With Autosomal Recessive Glycogen Storage Disease Type IV due to Complete Maternal Isodisomy of Chromosome 3

**DOI:** 10.1155/crig/5577571

**Published:** 2025-08-27

**Authors:** Sigrid Skovby Olsen, Anja Ernst, Pia Sønderby Christensen, Ellen Dagmar Björnsdóttir, Lasse Ringsted Mark, Albert Vejlin Stefansen, Allan Thomas Højland

**Affiliations:** ^1^Department of Clinical Genetics, Aalborg University Hospital, Aalborg, Denmark; ^2^Department of Molecular Diagnostics, Aalborg University Hospital, Aalborg, Denmark; ^3^Department of Pediatrics, Aalborg University Hospital, Aalborg, Denmark; ^4^Department of Pathology, Aalborg University Hospital, Aalborg, Denmark; ^5^Department of Clinical Medicine, Aalborg University, Aalborg, Denmark

**Keywords:** autosomal recessive, *GBE1*, glycogen storage disease type IV, GSD type IV, isodisomy, uniparental disomy

## Abstract

Uniparental disomy (UPD), the inheritance of two copies of a chromosome from one parent, can lead to recessive genetic disorders or imprinting effects. We report a case of autosomal recessive glycogen storage disease type 4 (GSD IV) due to maternal UPD of chromosome 3, representing the first reported instance of UPD leading to this rare disorder. To avoid an unjustified claim of misattributed paternity, the possibility of UPD should always be kept in mind in cases with the unique finding of the homozygous pathogenic variant only present in one parent. This case highlights the critical role of genetic counseling in uncovering rare genetic conditions and emphasizes the need for continued awareness of UPD in clinical genetics.

## 1. Introduction

Glycogen storage disease type 4 (GSD IV) (OMIM 232500) is a rare autosomal recessive disorder caused by pathogenic mutations in the *GBE1* gene located on chromosome 3. The *GBE1* gene encodes the glycogen branching enzyme, which catalyses the last step in glycogen biosynthesis with the transfer of alpha-1,4-linked glucosyl unit from the outer end of the glycogen chain to an alpha-1,6 position on a glycogen chain. A defect in the enzyme causes the production of glycogen with fewer branching points, making it less soluble, resulting in its accumulation in various organs including the liver, cardiac, and skeletal muscles as well as within the central nervous system. The clinical presentation of the disease is highly variable, ranging from fatal perinatal disease and uterine death to a milder disease with onset in the 2nd decade of life. GSD IV is rare, with an overall incidence of approximately 1:600,000–1:800,000, accounting for roughly 3% of all glycogen storage diseases [1]. Most pathogenic variants reported are missense mutations, where the patient is homozygous or compound heterozygous [2].

Uniparental disomy (UPD) is a rare condition and is defined as the inheritance of two copies of a given chromosome from one parent and none from the other parent [3]. UPD can be classified into uniparental isodisomy (iUPD) with two identical copies of a chromosomal segment and uniparental heterodisomy with two nonidentical copies of a chromosomal segment. Furthermore, UPD is classified according to whether the chromosome pair is of maternal or paternal origin. UPD causes increased risk of genetic disorders due to risk of recessive genetic disorders or due to imprinting effects [4]. UPD often arises due to an error in cell division during meiosis, resulting in abnormal transmission of chromosomes to the gamete. The abnormal gametic formation can result in the formation of a zygote with either monosomy or trisomy which can lead to monosomic or trisomic rescue in the zygote causing UPD. Furthermore, UPD can also be caused by mitotic errors [5].

To date, no cases of GSD IV caused by UPD have been reported. We report here on the first patient born with GSD IV due to iUPD.

## 2. Case Report

The patient was born prematurely at gestational age 35 weeks and 6 days to a 28-year-old woman (gravida 2, para 0), following a noncomplicated pregnancy with normal findings during routine prenatal ultrasound scans. The parents were nonconsanguineous. The child was delivered via spontaneous vaginal delivery. The newborn appeared severely hypoxic with an Apgar score of 1 at 5 min of age and 4 at 10 min of age. The infant was not breathing and was intubated at 9 min old. On clinical examination the infant was unresponsive and hypotonic with closed eyes and no movements. His clinical state indicated moderate encephalopathy due to a lack of the Moro, sucking, and grabbing reflexes. Blood gas analysis showed respiratory acidosis compatible with hypoventilation after delivery as well as hypoglycemia (glucose 1.4 mmol/L) despite glucose infusion. The initial biochemical workup was otherwise unremarkable, with no indication of alternative reversible aetiologies such as infectious disease or other metabolic causes. Severe hypoxic ischemic encephalopathy (HIE) was the main tentative diagnosis, but surprisingly the pH in the umbilical cord was normal with pH 7.26 and BE-5. Despite this and due to the time-sensitive nature of HIE, treatment with therapeutic hypothermia was initiated. At age 2 h, the newborn developed a seizure and received treatment with phenobarbital and midazolam. Lung and heart auscultations were normal, and no hepato- or splenomegaly was noted. Radiologic examination showed a small subdural hematoma without significance as well as diffuse changes compatible with ischemia, but also prematurity. The neonate was admitted to the ICU, where he received supportive treatment as well as antibiotics and antiviral medications. The neonate was cooled for 72 h. After rewarming, his cardiovascular state improved, and he was able to receive tube feeding. The MRI performed at 4 days of age did not support HIE. During the following days he showed alarmingly few signs of consciousness and very few movements. His eyes were open for short periods of time and were flaccid with little to no movement. He was able to trigger the ventilator but did not have the strength to breathe on his own. Attempts to extubate were unsuccessful.

**Figure 1:**
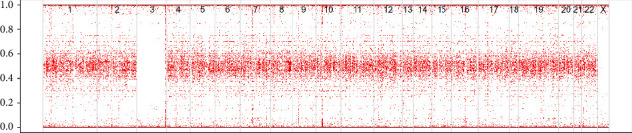
B-allele frequency (BAF) plot for WES data of the patient. The plot was constructed with ASCAT version 3.1.2 from Van Loo Lab Cancer Genomics Laboratory, The Francis Crick Institute [6]. The standard procedure for targeted WES sequencing data was followed and all settings for constructing the plot were kept as default. It can be observed that the B-allele frequencies for chromosome 3 are primarily clustered around 0 and 1, indicating the lack of heterozygosity for this chromosome.

Trio whole exome sequencing (WES) of the index and both parents was initiated after genetic counselling of the parents the day after delivery. WES library preparation was made using the SureSelect CREv2 capture kit (Agilent Technologies, Santa Clara, CA, US), and sequencing was performed on NovaSeq 6000 (Illumina Inc., San Diego, CA, USA). The sequencing data were analyzed through the internal pipeline at the Department of Molecular Diagnostics, Aalborg University Hospital. This pipeline is based on the GATK Best Practices [6]. The reads were aligned to reference genome hg19 with bwa mem [7], and variants were called using GATK's HaplotypeCaller [8]. Furthermore, a B-allele frequency (BAF) plot was constructed using ASCAT v3.1.2 [6] with default settings for targeted WES analysis (Figure 1). Trio analysis was run in Varseq Software v2.3 (Golden Helix, MT, USA), with filtering designed to look for *de novo* variants, homozygous variants, compound heterozygous variants, heterozygous variants inherited from either of the parents, copy number variants, and variants with Mendelian inheritance error. Oligoarray was also performed with a normal result to rule out microdeletions and microduplications.

The analysis revealed that the child was seemingly homozygous for a pathogenic variant (NM_000158.4: c.143 + 1G > C) in the *GBE1* gene on chromosome 3. No copy number variants were identified using both oligoarray and whole exome data. The variant was classified as likely pathogenic (C4) according to ACMG guidelines [9]. The mother was heterozygous for this variant, while the father was wild type. Further analyses of chromosome 3 were conducted and revealed: (1) 207 variants were found in a homozygous state in the index, heterozygous state in the mother, and missing in the father. (2) No variants were found in a heterozygous state in the index and father and missing in the mother; hence, no variants on chromosome 3 were transmitted from the father to the index. (3) No *de novo* variants were found in the index. (4) Variants found in a heterozygous state in the mother were not found in a heterozygous state in the index. (5) Numerous variants inherited from the father were found on all other autosomes. (6) No copy number variants were identified on chromosome 3 in both oligoarray and CNV analysis of exome data. (7) No variants were found in a homozygous state in the index and missing in one of the parents on all other autosomes. These findings strongly suggest maternal iUPD3 as the cause of homozygosity for the pathogenic *GBE1* variant in the index. Genetic diagnosis was made on the 12^th^ day postdelivery. The clinical findings matched the genetic diagnosis, and to avoid any unnecessary and painful interventions on the patient, no further blood tests were performed to biochemically confirm the diagnosis. The genetic diagnosis in combination with the child's clinical condition led to the decision of discontinuing the treatment. On the 13^th^ day, supportive treatment was discontinued, and shortly thereafter the neonate passed away.

## 3. Discussion

In this case, the genetic diagnosis was delayed due to the unusual finding of only one of the parents carrying the pathogenic variant in the *GBE1* gene. This was in part done to ensure that an unjustified claim of misattributed paternity did not arise. Disclosing misattributed paternity can have a wide range of consequences, which have been widely discussed throughout time, as well as whether misattributed paternity should be disclosed in various settings [10–12]. The likelihood of non-Mendelian inheritance increases with lower carrier frequency in the population [13, 14]. Therefore, the possibility of UPD should always be kept in mind to avoid potentially damaging discussions for the family as well as the doctor–patient relationship.

Here, we report the first case of GSD IV caused by maternal iUPD of a pathogenic variant in the *GBE1* gene, as determined by trio WES. Cases of UPD, especially with chromosomes that do not undergo genomic imprinting, may be underreported, as it previously has been technically difficult to identify. However, with improving technology, the true frequency of autosomal recessive diseases, caused by UPD, might be revealed. The natural frequency of UPD, as well as the recurrence risk of UPD, is unknown [14] but diagnosis of an autosomal recessive disorder due to UPD has been reported to decrease the recurrence risk from 25% to much lower.

## Data Availability

Data are available on reasonable request.
